# PERCEIVED DISCRIMINATION ON DEPRESSION IN OLDER ADULTS: MODERATING EFFECT OF GEOGRAPHIC ACCESS TO SOCIAL SERVICES

**DOI:** 10.1093/geroni/igae098.3684

**Published:** 2024-12-31

**Authors:** Seo-Yun Choi, Soondool Chung

**Affiliations:** University of Southern California, Los Angeles, California, United States; Ewha Womans University, Seoul, Republic of Korea

## Abstract

Age discrimination against older adults represents a significant issue in South Korea, with notable implications for their mental health and well-being. Addressing ageism is critical as it limits resources for detecting and treating depression in older adults, affecting their health behaviors and willingness to seek help. On the other hand, despite a high prevalence of depression among this population, only 2.2% utilize mental health services. This study examined how geographic accessibility to social services for diagnosing and treating depression can improve mental health service utilization among older adults experiencing age discrimination. About 9,920 individuals 65 and above from the National Survey of Older Persons were included. It explored the moderating effect of geographic accessibility on the relationship between perceived age discrimination and depression. Correlation and moderating effect analyses using SPSS PROCESS Macro Model 1 were computed, and the results demonstrated that perceived ageism is positively associated with depression (r=.220, p<.01). More importantly, geographic access to social services significantly moderated this relationship (B=-.180, p<.05), reducing the impact of perceived ageism on depression. These findings underscore the importance of enhancing geographic access to social services to mitigate the effects of ageism on depression among older adults. The study recommends direct measures, such as expanding social service infrastructure in rural areas, and indirect measures, like improving public transportation connectivity and introducing age-friendly technology. Further research will be necessary to understand regional characteristics better and enhance access to adequate social services.

